# Outcomes of surgical management of peptic ulcer perforation using the falciform ligament: A cross-sectional study at a single centre in Vietnam

**DOI:** 10.1016/j.amsu.2021.102477

**Published:** 2021-06-16

**Authors:** Tran Que Son, Tran Hieu Hoc, Tran Thu Huong, Vu Duc Long, Tran Thanh Tung, Nguyen Chien Quyet, Lun Panha, Nguyen Van Chi

**Affiliations:** a Department of Surgery, Hanoi Medical University, Viet nam; b Emergency Center (A9), Bachmai Hospital, Viet nam; c Department of Pharmacy, Bachmai Hospital, Viet nam

**Keywords:** Falciform ligament, Round ligament, Flap, Peptic ulcer, Perforation, Mortality

## Abstract

**Introduction:**

Peptic ulcer perforation (PUP) is one of the most common critical surgical emergencies. The omentum flap is commonly used to cover a PUP. However, the omentum cannot be used in cases of severe peritonitis or previous surgical removal. This is the first study conducted in Vietnam that was designed to analyse the outcomes of patients with PUPs who were treated using the falciform ligament.

**Method:**

In this study, we retrospectively identified 40 consecutive patients who were treated for PUP at a single high-volume centre in Vietnam from February 2018 to February 2021. Peptic ulcer perforation was measured during diagnostic evaluation based on preoperative imaging, such as X-ray, and CT scan. Patients who had malignancy, laparoscopic surgery, omentopexy and nonoperative treatment were excluded from this research.

**Results:**

Forty patients were included; the mean age of the patients was 66.3 years (range 33–99 years), and some patients had comorbid disease (57.5%), hypertension (30%), diabetes (10%), cirrhosis (7.5%), and chronic renal failure (7.5%). The PUPs were located in the duodenum (80%), or the pyloric (15%) and prepyloric (5%) regions. The procedures used to treat the patients included duodenostomy (32.5%), gastrojejunostomy (37.5%), and antrum resection (2.5%). The average operative time was 88.6 min (45–180 min), hospital stay was 9.6 days (2–35 days), and oral intake was started at 4.1 days (3–8 days); additionally, the 30-day mortality (17.5%) and incidences of pneumonia (25%), multiorgan failure (15%), acute liver failure (5%), wound infection (7.5%), and ulcer peptic fistula (0%) were assessed. Univariate tests showed that an ASA ≥ III and comorbidities, such as pulmonary complications, liver failure and multiorgan failure, were associated with mortality. The multivariate test showed that multiorgan failure was the only factor related to mortality.

**Conclusion:**

The falciform ligament can be efficiently used for the closure of a PUP. Although there were no instances of complication with a reperforated peptic ulcer, the mortality rate was slightly highly related to severe comorbidities and postoperative multiorgan failure.

## Highlights

• Peptic ulcer perforation is one of the most common surgical emergencies with an overall mortality rate is between 1.3% and 20%.
• Comorbidities, over 70 years old, having surgical therapy after 36 hours, and postoperative complications are associated with mortality.
• Using falciform ligament to replace the traditional omental patch is interesting, easy applying and efficiently in the closure of PUP.
• The mortality and complications were still high related to severe comorbidity and multiorgan failure after surgery.

## 1 Introduction

In the population, the prevalence of peptic ulcer disease based on physician diagnosis ranges from 0.12% to 1.50%, and hospitalization rates range from 0.10% to 0.19% [1]. Peptic ulcer perforation (PUP) is one of the most common surgical emergencies worldwide, and surgery should be performed as soon as possible. The overall mortality rate of PUPs is between 1.3% and 20%, and mortality due to a perforation of a stomach ulcer is higher than mortality due to a perforation of a duodenal ulcer (40% vs 10%) [2,3]. Some studies have shown that having comorbidities, being over 70 years old, undergoing surgical treatment after 36 h, PUP diameter greater than 1 cm^2^ and postoperative complications are related to mortality [4]. Therefore, technical improvements to decrease the complications of PUPs are one of the most important goals in treatment [5].

Depending on the clinical condition and PUP characteristics observed during each surgery, appropriate management methods should be used. Currently, laparoscopic surgery is the first choice when patients arrive early and have premature ulcers, and this technique is easy to perform. However, for patients who have undergone previous abdominal surgeries, and have large PUPs located in the pylorus or have suspected malignant ulcer perforations, laparoscopy procedures should not be performed, and the treatment should change to open laparotomy [6].

The use of the omentum to cover a PUP was introduced by Roscoe R. Graham in 1937 and is still widely used today to reduce the morbidity and mortality rate [7]. However, the greater omentum cannot be used in cases of severe peritonitis or previous surgical removal. On the other hand, dilated small intestine and colon due to peritonitis and abdominal distention cause omentum stretch, which results in a high (8) risk of punctures and leaks [2].

The technique of using the falciform ligament for the treatment of PUPs was reported in 1978 by Fry [8]. To date, there are few reports in the public literature regarding this technique. The use of the falciform ligament is effective in the repair of the PUP during open surgery as well as laparoscopic surgery, even if there is a large perforation with a size between 2 and 3 cm [[9], [10], [11]]. The purpose of the study was to describe the technique and the results of using the falciform ligament for the treatment of PUPs in a large volume surgical centre in Vietnam.

## 2 Methods

We performed a retrospective observational study that included all consecutive adult patients who underwent PUP surgery at the Department of Emergency Centre and General Surgery, Bach Mai Hospital between February 2018 and February 2021. This study is reported in accordance with the STROCSS 2019 criteria [12].

Written informed consent was obtained from all the patients before participation, and ethics approval was obtained from the Human Subjects Protection Committee of Bach Mai Hospital (126/QĐ-BM (01/17/2019)) and was signed by the Director of Bach Mai Hospital. The research registration unique identifying number (UIN) is research registry6771, and the study is available at researchregistry.com.

### 2.1 Antibiotics were started preoperatively according to the institutional protocol

This treatment included a third-generation cephalosporin (1 g of Basultam or 1 g of Sulperazon) plus metronidazole intravenously for at least 5 days. In complicated cases, antibiotics were changed based on the sensitivity results.

### 2.2 Protocol

Laparotomy with a midline abdominal wall incision was performed in all the patients.

The abdominal fluid was collected to submit bacterial cultures to determine the appropriate antibiotic treatment, and the abdominal cavity was flushed with 0.9% saline solution. The abdominal cavity and its contents, including the diaphragm, liver surface, and gallbladder, were evaluated. The stomach, duodenum, small intestine, colon, and Douglas were also evaluated.

Dissection of the falciform ligament was initiated near the umbilicus, and then the ligament was transected from the anterior abdominal wall to the diaphragm.

**Fig. 1:**
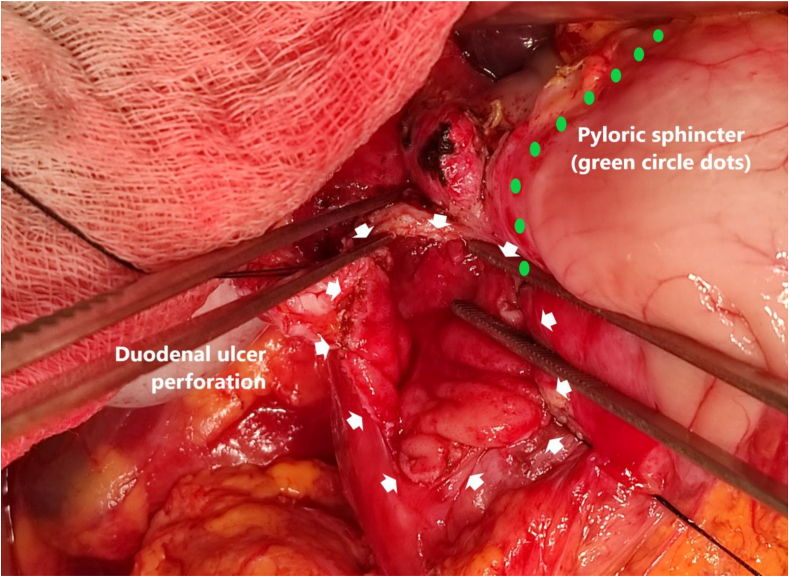
Duodenal ulcer perforation. The duodenal ulcer perforation was located at D1 (white arrow) below the pyloric ring (blue dot) and measured approximately 10 mm. The unhealthy tissues of the perforated ulcer were dissected to the healthy duodenal wall and then closure of the perforated ulcer was performed with a vertical incision and stitched horizontally. (For interpretation of the references to colour in this figure legend, the reader is referred to the Web version of this article.)

**Fig. 2:**
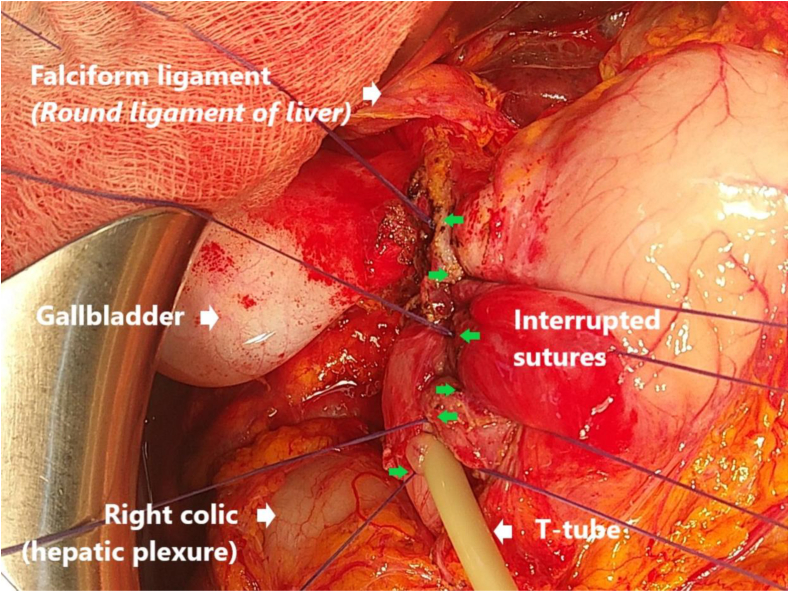
A T-tube was placed and closure was obtained with full-thickness simple interrupted sutures using only safil 2–0 or 3–0. The T-tube size was 14 or 16 Frankel and was used to drain biliary and pancreatic fluid out of the body. This drainage was allowed to flow freely for 7–14 days, and was withdrawn after 30 days.

Biopsies were taken from the ulcer edge for pathological analysis (Fig. 1) Interrupted sutures were placed through all the layers of the peptic ulcer with either safil or vicryl 3–0 (B. Braun Aesculap AG&CO.KG, Spain) separate sutures (Fig. 2). In some cases, the peptic ulcer diameter was more than 2 cm, or a pyloric perforation was observed. Therefore, a T-tube of size 14 or 16 Fr was placed at the duodenum (Fig. 2).

**Fig. 3:**
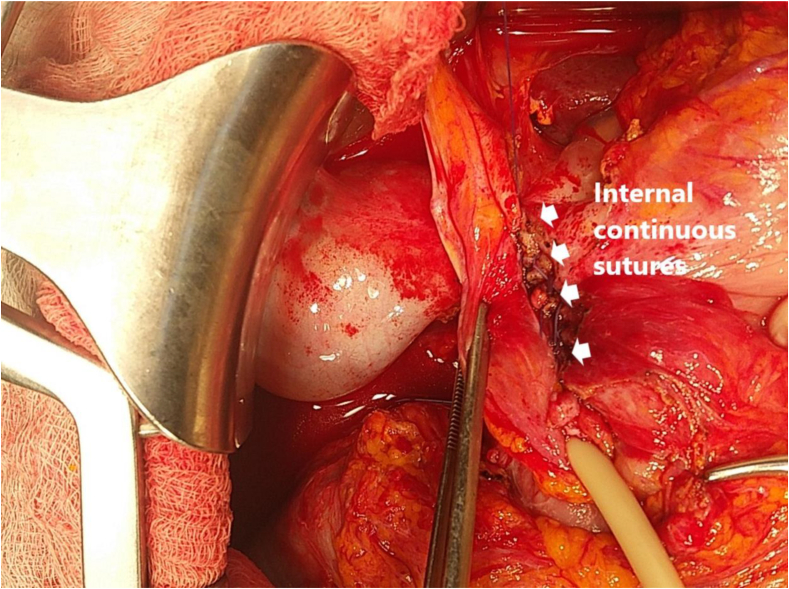
Internal layer with a continuous suture using safil 3–0 connecting the duodenal seromuscosa below the ulcer to the falciform ligament.

**Fig. 4:**
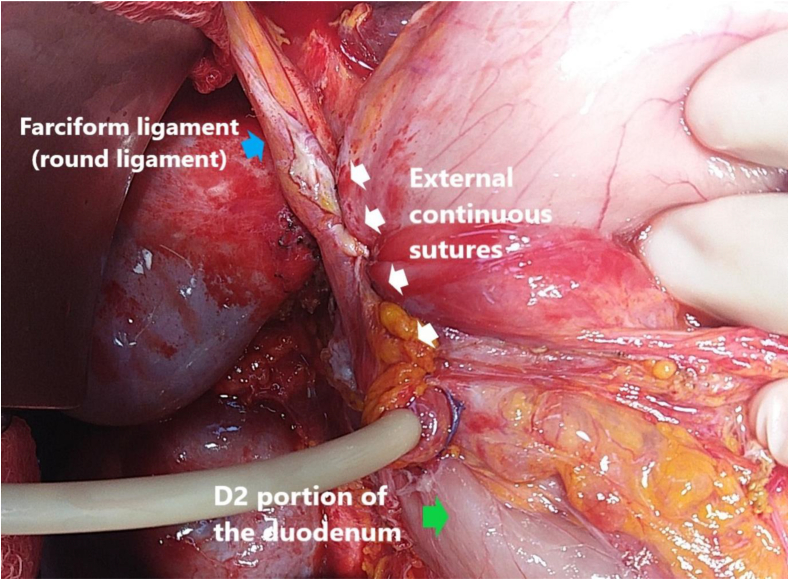
Outer layer with a continuous suture (white arrow) the seam of the duodenal perforation.

In the inner region, the anterior wall below the edge of the ulcer was sutured with a simple continuous pattern with safil 4–0 (B. Braun Aesculap AG&CO.KG, Spain) and was continued to the falciform ligament (Fig. 3). Then, the wall upper ulcer to the ligament in the outer surface was sutured with a continuous pattern (Fig. 4).

We used common sutures with most of the PUPs in the D1 location with small diameters. We sutured and inserted a T-tube in case of a strong edge or a large PUP with a size more than 1 cm or with PUPs in pre-pyloric or pyloric locations. For antrum resection, we drained the duodenum with Petzer and sutured the falciform ligament around the drain in cases of large PUPs. Gastrojejunostomy should be performed when the risk of duodenal stenosis or the side of the peptic ulcer is located near the pylorus.

### 2.3 Follow-up

The nasogastric tubes were removed from patients after 3–4 days. On postoperative day 4 or 5, the patients were started on oral fluids. Postoperative antibiotic treatment was continued for 7–10 days, and proton pump inhibitors were continued for 1 month. Postoperative intensive care was provided as indicated based on the institutional protocols.

### 2.4 Outcome measures

The perioperative data, including history and examination findings, were recorded, and preoperative laboratory test variables, treatment related variables, specimen related variables, and postoperative complications were recorded for analysis. Postoperative mortality was defined as the death of the patient in the hospital during the same admission period. Postoperative complications, such as wound infection, chest infection, renal failure, cardiac failure, septic shock, or the need for mechanical ventilation during the same admission period, were analysed.

### 2.5 Statistical analysis

Data in this study were analysed using SPSS 20.0 software (IBM, USA). For quantitative variables, the mean was compared by an independent sample Student's t-test or the Mann-Whitney *U* test. For qualitative data, the chi-square test or Fisher's precision test was used. The data are presented as the mean ± standard deviation (SD). The statistical tests were considered statistically significant when p < 0.05.

All the data were analysed by univariate analysis, and only variables with p < 0.05 were included in multivariate logistic regression analysis to identify independent risk factors for postoperative mortality. The results are presented as their odds ratio (OR) and 95% confidence intervals (CI).

## 3 Results

A total of 40 patients underwent surgery. The mean age was 66.3 years (ranging from 33 to 93 years); 57.5% of patients had comorbidities, 30% had hypertension, and some patients had a history of peptic ulcer (17.5%), cardiovascular disease (17.5%), or severe cirrhosis (7.5%). Blood test results were within the normal range (Table 1). The most common location of ulcer perforation was the duodenum (80%), followed by the pyloric region (15%). Surgical management included inserted duodenal drainage (32.5%), anterior colonic mesenteric gastrojejunostomy (37.5%), and antrum resection (one patient). The average hospital stay was 9.6 days (range from 2 days to 35 days). Oral intake was initiated at an average of 4.1 days.

**Table 1:** Patients’ background.

Characteristics	Number of patient (n = 40)
Age, median (range) [year]	66.3 (33–93)
Gender, n (%)	
Male	26 (65)
Female	14 (35)
Comorbidity, n (%)	23 (57.5)
Diabetes melilitus, n (%)	4 (10)
Peptic ulcer, n (%)	7 (17.5)
Hypertension, n (%)	12 (30)
Cardiovascular disease, n (%)	7 (17.5)
Cirrhosis, n (%)	3 (7.5)
Chronic kidney disease, n (%)	3 (7.5)
Brain strock, n (%)	4 (10)
WBC, median (range) [G/L]	13.39 (1.3–69.2)
RBC, median (range) [T/L]	4.31 (1.38–5.49)
Prothrombin, median (range)	86.8 (42–137)
Creatinin, median (range) [μmol/l]	94.8 (33–243)
Albumin, median (range) [g/L]	34.3 (21–42)
PLT, median (range) [G/L]	293.25 (36–521)
WBC: White blood cell, RBC: red blood cell, PLT: Platalet count	

Pneumonia was the most common complication (25%), followed by multiorgan failure (15%), and liver failure (5%). In particular, there were no cases of gastrointestinal leakage (0%) or reoperation (0%). The mortality was slightly high at 17.5% (Table 2). Univariate analysis showed several factors related to postoperative mortality, such as ASA ≥ III, comorbidities, pulmonary complications, liver failure and multiple organ failure (Table 3). Multivariate analysis showed that multiorgan failure was the only factor associated with postoperative mortality (p = 0.043, OR = 1.499) (Table 4).

**Table 2:** Localization of peptic ulcer and results of operative technique.

Characteristics	Patient (n = 40)
Site of perforation	
Duodenum, n (%)	32 (80)
Pre-pyloric, n (%)	2 (5)
Pyloric, n (%)	6 (15)
Duodenostomy	
T-tube, n (%)	9 (22.5)
Petzer, n (%)	4 (10)
Gastrojejunostomy, n (%)	15 (37.5)
Antrum resection, n (%)	1 (2.5)
Operative time, media (range) [min]	88.6 (45–180)
Length of hospital stay, median (range) [days]	9.6 (2–35)
Oral intake, median (range) [days]	4.1 (3–8)
30-day mortality, n (%)	7 (17.5)
Leak, n (%)	0 (0)
Ileus, n (%)	1 (2.5)
Evisceration, n (%)	1 (2.5)
Pneumoniae, n (%)	10 (25)
Wound infection, n (%)	3 (7.5)
Acute liver failure, n (%)	2 (5.0)
Multi organs failure, n (%)	6 (15)

**Table 3:** Univariate logistic regression analysis for mortality.

Factors	All patients (n = 40)	Non-mortality (n = 33)	Mortality (n = 7)	P
Age	66.23 ± 15.2	64.88 ± 14.97	72.57 ± 15.82	0.631^ǂ^
≤67	22	18	4	1,000^ƚ^
>67	18	15	3	
ASA				
≤ II	24	23	1	0,001^ƚ^
≥ III	16	10	6	
Comorbidity				
Yes	23	16	7	0,014^ƚ^
No	17	17	0	
Hypertension				
Yes	12	12	0	0,081^ƚ^
No	28	21	7	
Cirrhirosis				
Yes	3	1	2	0,074^ƚ^
No	37	32	5	
Diabetes				
Yes	4	2	2	0,134^ƚ^
No	36	31	5	
Chronic kidney disease				
Yes	3	1	2	0,074^ƚ^
No	37	32	5	
Ulcer's diameter				
≤1 cm	32	26	6	1,000^ƚ^
>1 cm	8	7	1	
Duodenostomy				
T-tube	9	8	1	0,667^ƚ^
Petzer	4	3	1	
Non	27	22	5	
Gastrojejunostomy				
Yes	15	11	4	0,392^ƚ^
No	25	22	3	
Antrum resection				
Yes	1	1	0	1,000^ƚ^
No	39	32	7	
Pulmonary complications				
Yes	10	5	5	0,006^ƚ^
No	30	28	2	
Liver failure				
Yes	2	0	2	0,027^ƚ^
No	38	33	5	
Multiple organ failure				
Yes	6	2	4	0,005^ƚ^
No	34	31	3	

All results were presented as n or mean ± SD as appropriate. ‡Mann-Whitney *U* test; †Chi-squared test.

**Table 4:** Multivariate logistic regression analysis for mortality.

Variable	B	S. E	Beta	Sig.	Exp(B)	95% CI^¥^	
						Lower	Upper
ASA	0.080	0.154	0.103	0.609	1083	0,791	1477
Comorbidity	0.126	0.121	0.164	0.302	1134	0,887	1449
Pulmonary complications	0.298	0.149	0.340	0.052	1347	0,996	1822
Acute Liver Failure	0.276	0.283	0.159	0.336	1317	0,741	2344
Multi Organ Failure	0.392	0.186	0.368	0.043	1499	1150	2157

¥ Durbin – Watson test, B: regression coefficient; Sig: P value; Exp(B): OR.

## 4 Discussion

Peptic ulcer perforation is one of the most serious surgical complications that can lead to death. Mortality from PUP surgery ranges from 2 to 22% and contributes to 37% of all peptic ulcer-related deaths [6]. Imhof's study on 108 patients with duodenal ulcer perforation showed that these patients had a poor prognosis, with mortality rates after 1 month, 1 year, and 5 years of 9.1%, 20.2% and 32.3%, respectively. The multivariate analysis revealed several factors that were related to mortality, such as comorbidities, postoperative complications and old age [5].

Questions related to the management of a PUP were as follows [1]: Is surgery indicated? [2] Is an omental patch indicated as sufficient for ulcer operation ? [3] Is the patient able enough to undergo surgery? [4] Should surgerycompletely remove the ulcer? [5] If newer methods affect the outcome of treatment, should surgery be indicated ? And [6] Should laparoscopic surgery be indicated? Some methods are used, such as simple perforation sutures, that can suture the PUP and cover it with the omentum (Cellan-Jones repair) or with Graham patches [3]. One of the most widely adopted methods is Graham's patch technique. According to the first description of this technique by Graham, several interrupted sutures are taken through the defect untied; then, the greater omentum is placed between these sutures; and finally, the sutures are tied to hold the omentum in the perforated ulcer. For the success of this technique, the omentum should be viable and not strangulated [13]. This method should only be applied to small perforations of less than 1 cm in diameter to ensure that the repair is strong and leakage does not occur. Several other modifications of the Graham technique have been reported [10,13]. To date, the Graham patch has been widely adopted worldwide because it is simple, easy to implement and effective in treatment [9]. However, the omentum of thin or elderly patients may be very attenuated or virtually non-existent. However, in some select patients where the greater omentum is either unviable, unhealthy, or cannot be utilized, the falciform ligament can be used as an adequate patch for closure [2,13].

The falciform ligament consists of 2 layers of the peritoneum, and the membrane consists of two parts: the membranous part and fatty part. The falciform ligament includes ligamental teres, paranasal veins (paraumbilical veins) and fatty parts. Arteries are present from the arterial branches of the left diaphragm and between liver lobes. Venous blood drains from the left diaphragmatic vein and portal vein. Ligament teres (or round ligaments) are relics of the foetal umbilical vein, draining blood to the left portal vein. In adults, the round ligament is located at the edge of the free ligament (falciform ligament). Therefore, the falciform ligament has a similar hypervascular omentum [14,15]. In addition, the falciform ligament can also be flexibly used to repair damage to the bile ducts; cover a diaphragmatic hole, create an anti-oesophageal reflux valve, surround the outside oesophageal anastomosis after total gastrectomy, cover the gallbladder bed to stop haemostasis or prevent bleeding and bile leakage; it can even be used to form artificial arteries [[16], [17], [18], [19]]. In the undisturbed state, the falciform ligament lies across the first part of the duodenum and can be sutured to an ulcer without tension or mobilization to increase strength and nourishment to the main peptic ulcer region [20].

In the literature, there is little research on applying falciform ligaments in the treatment of PUP, but most are clinical case reports with positive results (Table 5). Fry et al. (1987) was the first to use a flap of the falciform ligament to repair a PUP. With adequate mobilization, the falciform ligament can serve as a viable pedicle to achieve closure of perforations in the first portion of the duodenum [8]. The middle segment artery of the liver and the left phrenic artery provide the main blood supply of the falciform ligament, making it a well-vascularized structure to be used as a flap [15].

**Table 5:** Literature review using falciform ligament for peptic ulcer perforation.

Author	Year	Article type	Number of patients (n)	Indication	Site and ulcer size (mm)	Method	Failure n (%)
Fry DE	1978	Case	1	Poor omentum	Duodenum [15]	Open	0
Costalat	1995	Retrospective	12	New technique	NA	Open	0
Munro	1996	Case	6	New technique	Duodenum (NA)	Open	0
Bingerner	2013	Case	1	New technique	Duodenum (NA)	Open	0
Boshnaq	2016	Case	1	Pan-proctocolectomy	Pre-pyloric (30)	Open	0
Allart	2018	Case	1	New technique	Duodenum (NA)	Lap	0
Olmez	2019	Series case	46	New technique	Duodenum [5]	Open	4 (8.7%)
Ahmadinejad	2020	Case	1	New technique	Stomach curvature [20]	Open	0
Elgazar A	2020	Case	1	New technique	Duodenum [20]	Open	0
Takahashi Y	2020	Case	1	Previous omentectomy	Duodenum [5]	Open	0
This study	2021	Series case	40	New technique	Peri-pyloric (10.3)	Open	7 (17.5)

NA: not available.

Costalat (1995) aimed to apply a laparoscopic endoscopic technique in 15 PUP cases using ligamental teres hepatitis for the repair of an anterior perforated duodenal ulcer diagnosed within the previous 6 h. The procedure could not be performed in three patients: in one patient, the diameter of the perforation exceeding 15 mm, and two patients had severe peritonitis. This technique should be considered for young patients in whom the ulcer has been complicated by a fresh perforation [21].

Munro et al. (1996) used the falciform ligament in the repair of a PUP. The falciform ligament lies across the first part of the duodenum and can be sutured to an ulcer without tension or mobilization. All six patients were discharged by day 5, and no complications were reported. The authors found that the falciform ligament was an excellent, simpler alternative, especially in laparoscopic surgery [20].

A prospective pilot study by Bingener et al. (2013) was attempted using the NOTES technique using hepatic ligamental teres for one patient. This approach was more appropriate than using omentum and did not result in any leakage [21,22].

Boshnaq et al. (2016) reported a case of an 83-year-old woman in whom a perforated prepyloric ulcer was closed using a falciform ligament pedicle flap due to the absence of omental fat, likely due to a previous panproctocolectomy [23].

Aydemir Ölmez (2019) reported a study comparing the retrospective results of patients who underwent falciformopexy or omentopexy for PUP and showed that there was no difference in morbidity and mortality between the two groups [11].

Takahashi (2020) reported a case of duodenal perforation in one patient who underwent resection of the total omentum, uterus and ovaries caused by metastatic ovarian cancer. Use of the falciform ligament was a suitable option, even in laparoscopic surgery [24]. To the best of our knowledge, laparoscopic repair is more commonly utilized for low- or medium-risk patients, and open repair is chosen for the more at-risk patient population.

Most authors have stated that the omentum is more effective than the falciform ligament due to its ability to retain leaks, adhesiveness, lymphocyte rich vascular supply and ability to adhere to the area of inflammation. Falciform ligament or ligament teres should be preferred in cases where the omentum cannot be used [11,21,22].

In clinical practice, we removed unhealthy peptic ulcers and then closed the PUP with safil 2–0 or 3–0 (B. Braun Aesculap AG&CO.KG, Spain). In some cases, the PUP was drained out, and gastrojejunostomy was performed. To the best of our knowledge, using a falciform ligament to cover the PUP, as described here is an easy technique for effective application. To cover the PUP, we used two continuous sutures with inside stitches (Fig. 3) and outer stitches (Fig. 4), which can be wrapped around the duodenal drain. In this way, the falciform ligament was not constricted or strangulated but still covered the front of the peptic ulcer perforation. The average time to oral intake was 4.1 days (Table 2). In addition, 15 patients (37.5%) underwent anterior colic mesenteric gastrojejunostomy, and 13 patients (32.5%) underwent duodenostomy (Table 2). These techniques also reduced the risk of re-perforation by reducing the flow of digestive secretions through the duodenum. Although no cases of gastrointestinal leaks have been reported or reoperated after surgery, the effectiveness of using the falciform ligament to cover PUPs needs to be further studied. This method can be used to replace the omentum to reduce reoperation for PUP.

The technique of using a falciform ligament pedicle flap is not the only alternative surgical approach currently utilized instead of the classical greater omental patch (Graham) technique. Depending on PUP characteristics, such as location, and diameter size, patient's condition, suspected malignancy, severe stomach bleeding, pyloric stenosis, and giant duodenal ulcer, other techniques need to be performed, such as a jejunal serosal patch, a duodenostomy and pyloric exclusion, a Roux-en–Y duodenojejunostomy or a subtotal gastrectomy [23].

This is a new technique that was performed in our hospital for the first time. This research has certain limitations. The number of patients was limited, and only open surgery was applied. Therefore, the difficulty of this technique in laparoscopic surgery has not been assessed. In addition, many combined techniques were also performed during surgery to reduce the high risk of duodenal ulcer perforation leakages, such as duodenostomy or gastrointestinal anastomosis. Therefore, it is necessary to conduct prospective comparative studies between the falciformopexy method and the omentumopexy method or between the falciformopexy technique and the single perforated suture method to determine the effectiveness of the technique that we adopted.

## 5 Conclusion

Using the round or falciform ligament to replace the traditional omental patch is interesting, easy to apply and can be efficiently used in the closure of perforated duodenal ulcers. However, mortality and post-operative complications are still associated with severe comorbidities and multiorgan failure after surgery.

## Ethical approval

Written informed consent was obtained from all patients before participation, and ethics approval was obtained from the Human Subjects Protection Committee of Bach Mai Hospital: 126/QĐ-BM (01/17/2019) was signed by the Director of Bach Mai Hospital.

## Sources of funding

None.

## Author contribution

Tran Que Son: study concept, data collection, data analysis & interpretation, writing the papers and main surgeon.

Tran Hieu Hoc: study concept, data collection, interpretation and writing the papers.

Tran Thu Huong: study concept, data analysis & interpretation, writing the papers and edit English language.

Nguyen Chien Quyet: participating in surgery, collecting data.

Lun Panha: participating in surgery, collecting data.

Tran Thanh Tung: study concept.

Vu Duc Long: Supervisor.

Nguyen Van Chi: supervisor. Registration of Research Studies.

## Research Registration number

1. Name of the registry: **researchregistry. com**
2. Unique Identifying number or registration ID: **researchregistry6771**
3. Hyperlink to your specific registration (must be publicly accessible and will be checked): https://www.researchregistry.com/browse-the-registry#home/registrationdetails/6086e0eb3e4b0d001dcb3ec6/

## Guarantor

The correspondences of this paper: Assoc. Prof. Ph.D. Tran Hieu Hoc.

## Consent

Written informed consent was obtained from the patient for publication of this case report and accompanying images. A copy of the written consent is available for review by the Editor-in-Chief of this journal on request.

## Provenance and peer review

Not commissioned, externally peer-reviewed.

## Declaration of competing interest

Authors declare no conflict of interest.
